# Transperineal prostate biopsy identifies locations of clinically significant prostate cancer in men considering focal therapy with PI‐RADS 3–5 regions of interest

**DOI:** 10.1002/bco2.111

**Published:** 2021-10-05

**Authors:** Nelson Stone, Vassilios Skouteris, Samuel Chang, Athanasios Klimis, M. Scott Lucia

**Affiliations:** ^1^ Department of Urology Icahn School of Medicine at Mount Sinai New York NY USA; ^2^ Hygeia Brachytherapy Center Hygeia Hospital Athens Greece; ^3^ Departments Radiology and Pathology University of Colorado Anschutz Medical Campus Aurora CO USA; ^4^ Department of Pathology Hygeia Hospital Athens Greece

**Keywords:** mpMRI, prostate biopsy, transperineal mapping

## Abstract

**Objectives:**

To determine the benefit of performing transperineal prostate mapping biopsy (TPMB) following multiparametric magnetic resonance imaging (mpMRI) to increase the identification of clinically significant prostate cancer (csPCa) with Gleason grade group (GG) ≥ 2 and their locations outside of the PI‐RADS v2 3–5 category lesions.

**Methods:**

mpMRI was performed in 80 men prior TPMB from two institutions. The mpMRI was considered clinically significant (csMRI) if it contained one or more PI‐RADS 3–5 category lesion. mpMRI findings were compared against csPCa diagnosed by TPMB, performed between 16 November 2010, and 13 September 2019, for the entire gland, both lobes and to the right and left anterior and right and left posterior quadrants (RA, LA, RP and LP). Sensitivity, specificity, positive and negative predictive values (PPV, NPV), accuracy and the area under curve (AUC) were determined. Thirteen men also underwent radical prostatectomy and had comparison of TPMB pathology to prostatectomy specimen grading.

**Results:**

TPMB was positive in 60/80 (75%) of which 32 (53.3%) were csPCa. csPCa was present in the RA in 9 (11.3%), LA in 11 (13.8%), RP in 25 (31.3%) and LP in 27 (33.8%) and involved 1 quadrant in 7 (21.9%), 2 quadrants in 12 (37.5%), 3 quadrants in 11 (34.4%) and all 4 quadrants in 2 (6.3%) patients; 57/80 (71.3%) men had a mpMRIs with lesions designated as PI‐RADS 3 in 24 (30%), 4 in 25 (31.3%) and 5 in 8 (10%). A csMRI was present in the RA in 7 (8.8%), LA in 8 (10%), RP in 31 (38.8%) and in the LP in 29 (36.3%), which were limited to one quadrant in 39 (68.4%), 2 quadrants in 16 (28.1%), and 3 quadrants in 2 (3.5%). Sensitivity, specificity, PPV, and NPV were determined from the results of the TPMB and were for the entire gland 81.3%, 35.4%, 45.6% and 73.9%. There were 31 csMRIs involving the right posterior of the gland but only 25 csPCa by TPMB of which 12/31 (38.7%) were concordant for high grade disease. There were 29 men who have a csMRI in the left posterior quadrant, and 14 (48.3%) were concordant with csPCa from the TPMB.

**Conclusions:**

MpMRI should be supplemented with TPMB to correctly identify the regions of the prostate that would require ablation in men considering focal therapy.

## 1 INTRODUCTION

The diagnosis of prostate cancer (PCa) is increasingly managed with the addition of multiparametric magnetic resonance imaging (mpMRI). An mpMRI study utilising the Prostate Imaging and Data Reporting System version 2 (PI‐RADS v2) criteria accurately identifies high grade PCa when a score of 5 is present.^1^ Although investigators report high negative predictive values (NPVs) for mpMRI when transrectal targeted and systematic biopsies are performed, the need for additional biopsy when no cancer is found in the gland outside the PI‐RAD 3–5 lesion is being questioned.^2^ More than 30% of clinically significant PCa (csPCa) lesions can be missed by mpMRI because of multifocality, lesion location, and size when compared with histopathology data of transperineal prostate mapping biopsy (TPMB) or whole‐mounted radical prostatectomy specimens (WMRPs).^1^, ^3^, ^4^, ^5^, ^6^

Physicians are increasingly interested in focal therapy, and more than 50% of urologists in a recent survey believed focal therapy is beneficial in the treatment of PCa, but 63.2% were also concerned that only treating the index lesion would be inadequate.^5^ Given that 70% or more patients clinically present with multifocal disease, it is understandable that practitioners would be reluctant to rely on an mpMRI to identify all csPCa lesions requiring ablation.^7^

TPMB has been shown to identify more csPCa than TRUS biopsy including the small lesions missed by mpMRI.^3^, ^8^ If targeted biopsy of mpMRI regions of interest (ROIs) with concomitant 12‐core systematic biopsy fails to identify csPCa outside of the ROI, then should that patient still be considered a candidate for focal ablation? While prostatectomy studies suggest that relying on the mpMRI to determine inclusion criteria for hemi‐ablation can be erroneous more than 50% of the time, this information is not helpful to the clinician who wants to make a shared decision about the optimal therapy before initiating treatment.^9^ We undertook this investigation to determine whether TPMB could improve patient selection for focal therapy by identifying csPCa outside of the quadrant detected by clinically significant MRI (csMRI). csMRI was defined as MRI lesions designated as PI‐RADS v2 3–5. In addition, this investigation sought to determine what quadrants of the prostate might be spared treatment when electing focal or subtotal therapy.

## 2 METHODS

Eighty patients from the University of Colorado Hospital (UCH, *n* = 65) and Hygeia Brachytherapy Center (HBC, *n* = 15) had an mpMRI prior to TPMB between 16 November 2010, and 13 September 2019. Eleven (13.8%) men were biopsy naïve, 18 (22.5%) were biopsy negative and 51 (63.7%) had a prior positive TRUS biopsy of which 37 (75.2%) had Gleason grade group (GG) 1, 12 (28.5%) had GG 2, 1 had GG 3 and 1 GG 5. The mpMRI and TPMB were compared to determine the presence, grade and location of the cancer and if the patient was a candidate for focal therapy. Subtotal treatment of the gland could be considered for clinically significant prostate cancer (csPCa), defined as GG ≥ 2, and occupying 3 (out of 4) or less quadrants.

**FIGURE 1:**
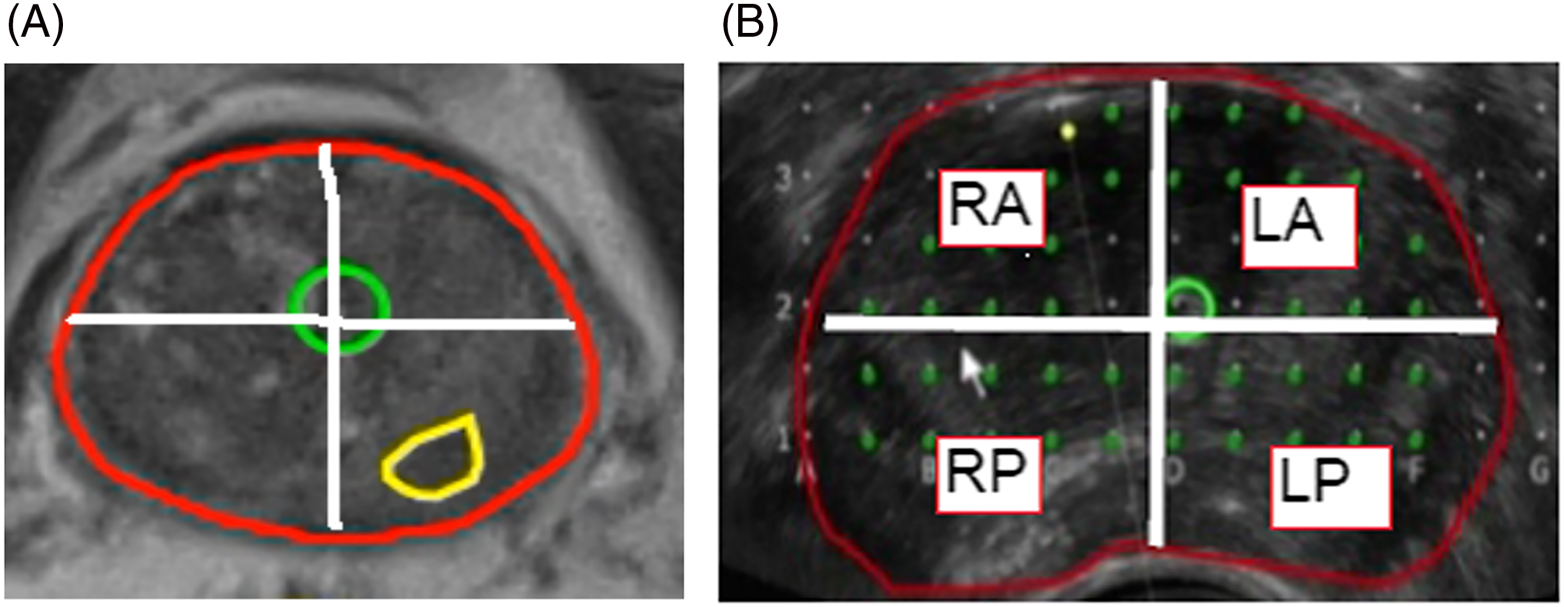
(A) T2 sequence of multiparametric magnetic resonance imaging (mpMRI) with prostate divided into 4 sectors with urethra (green circle) as axis. Region circled in yellow represents PI‐RADs 4 lesion. (B) Four sectors (quadrants) from axial ultrasound prostate image. Red line represents circumference of prostate and green circle urethra. Green dots are template puncture sites. RA—right anterior, LA—left anterior, RP—right posterior, LP—left posterior. Proprietary software programme not available for clinical use

At UCH, mpMRI was read by two experienced radiologists, and suspicious lesions were reported per PI‐RADS v2 criteria. mpMRI was performed with and without IV contrast on 3.0 Tesla scanner utilising an 8‐channel pelvis phased array surface or endorectal coil. mpMRI protocol included large and small field‐of‐view images with tri‐planar high resolution T2 images, diffusion weighting with ADC maps (*b* value of 0, 600 and 1000) and dynamic contrast. At HBC, mpMRIs were performed on 3.0 Tesla scanners with endorectal coils with contrast utilising the same sequences. The MR‐T2 images were loaded into proprietary software and ROIs were segmented (Figure 1A). The prostate was divided into right and left halves and four sectors, right (RA) and left (LA) anterior and right (RP) and left (LP) posterior with the urethra as the axis point (Figure 1B). If multiple lesions were present, each lesion (and respective sector) was noted. Lesions extending into contiguous sectors were counted as present in two or more regions. MRI lesions with a PI‐RADS score of 3–5 were considered csMRI.

TPMB was performed using the BK ProFocus with model number 8558/8568 transrectal probe (Peabody, MA, USA) under anaesthesia using an 18‐G disposable biopsy device through a 5‐mm template as previously described.^10^ A proprietary programme was used to create intraoperative 2D and 3D prostate images from the ultrasound and to record the location of the biopsy sites.^11^ Each specimen was analysed for the presence, length and location of PCa and assigned a GG from 1 to 5.^12^ All biopsy sites were numbered sequentially and matched to the same position in the software.

Thirteen men had radical prostatectomy surgery, and the final histopathology was compared with the mapping results to determine grading concordance between the two. Associations between GG and the number of positive biopsy sites were determined by analysis of variance (ANOVA) (bootstrapping). Contingency tables (Pearson chi‐square) were constructed for csPCa (derived from the TPMB) versus csMRI. Sensitivity, specificity, positive predictive value (PPV), NPV, accuracy and the area under the curve (AUC) were calculated for the entire prostate, both sides of the gland and for the four sectors. A csMRI confirmed by a csPCa from the TPMB was considered a true positive. The highest GG from the TPMB was compared with the highest GG from the prostatectomy specimen to determine grading accuracy. An increase by a GG of 1 or more was considered upgrading. Two‐way analyses with a significance ≤ 0.05 were performed using SPSS v.20. All data were de‐identified and anonymised and approved for reporting by the institutional review boards.

## 3 RESULTS

The median (range) patient age, prostate‐specific antigen (PSA) and prostate‐specific antigen density (PSAD) were 64.0 (46–82) years and 6.0 ng/mL (1–32) and 0.13 (0.019–1.1) (Table 1). TPMB was positive in 60/80 (75%) of which 32 (53.3%) were csPCa. There was no cancer in 20 (25%), GG 1 in 28 (35%), GG 2 in 21 (26.3%), GG 3 in 3 (3.7%), GG 4 in 5 (6.3%) and GG 5 in 3 (3.7%). cPCa lesions were more often bilateral when GG ≥ 2 was encountered (67.5%, OR 6.2, 95%CI 1.9–20.9, *p* = 0.002). csPCa was present in the RA in 9 (11.3%), LA in 11 (13.8%), RP in 25 (31.3%) and LP in 27 (33.8%). By quadrant, csPCa was localised to 1 quadrant only in 7 (21.9%), 2 quadrants in 12 (37.5%), 3 quadrants in 11 (34.4%) and in all 4 quadrants in 2 (6.3%); 30/32 (93.8%) men had csPCa isolated to 3 or fewer quadrants; 9/32 (28.1%) men had csPCa limited to one side of the prostate. The mean (range) number of cores containing csPCa by quadrant location were RA 9.2,^5^, ^6^, ^7^, ^8^, ^9^, ^10^, ^11^, ^12^, ^13^, ^14^, ^15^, ^16^, ^17^, ^18^, ^19^ LA 9.9,^2^, ^3^, ^4^, ^5^, ^6^, ^7^, ^8^, ^9^, ^10^, ^11^, ^12^, ^13^, ^14^, ^15^ RP 8.8 (2–29) and LP 10.7 (4–29); 9/32 (28.1%) men potential candidates for focal therapy had csPCa limited to GG 2–3 and 6 or fewer positive cores with the disease localised to 1 quadrant in 4, 2 quadrants in 3 and 3 quadrants in 2. Increasing the number of positive cores to 10 yielded for partial gland ablation consideration yielded 14/32 (43.8%) candidates.

**TABLE 1:** Patient and TPMB characteristics in 80 men who had mpMRI

Variable	Mean	Median	SD +	Minimum	Maximum
Age (years)	65.2	64.0	8.3	46	82
PSA (ng/mL)	6.2	6.0	4.3	1	32
Prostate volume (cc)	45.2	41.4	17.2	19	105
PSAD	0.16	0.13	0.16	0.019	1.1
Number cores	69.8	62.5	30	24	169
Biopsy density	1.6	1.6	0.47	0.6	2.7
Positive cores left gland	4.2	4.0	4.0	0	17
Positive cores right gland	5.5	5.0	3.0	1	12
Positive cores total	9.6	9.3	5.7	2	29

*Note*: 60 (75%) had prostate cancer, and 32 (53.2%) were clinically significant. Biopsy density was the ratio of the number of cores (specimens) to the prostate volume.

Abbreviations: mpMRI, multiparametric magnetic resonance imaging; PSA, prostate‐specific antigen; PSAD, prostate‐specific antigen density; TPMB, transperineal prostate mapping biopsy.

Fifty‐seven of 80 (71.3%) men had csMRI with PI‐RADS lesions designated as 3 in 24 (30%), 4 in 25 (31.3%) and 5 in 8 (10%). A csMRI was present in the RA in 7 (8.8%), LA in 8 (10%), RP in 31 (38.8%) and in the LP in 29 (36.3%) and limited to one quadrant in 39 (68.4%), 2 quadrants in 16 (28.1%) and 3 quadrants in 2 (3.5%). Forty‐one (71.9%) had a csMRI limited to one side of the prostate. Of the 31 csMRIs involving the right posterior of the gland and 29 in the left posterior quadrant, only 12 (38.7%) on the right and 14 (48.3%) on the left were concordant with the TPMB for csPCa (Table 2). Sensitivity, specificity, PPV and NPV for the entire gland were 81.3%, 35.4%, 45.6% and 73.9%. Accuracy and the AUC were 53.8% and 0.583 (95% CI 0.457–0.709) (Table 3).

**TABLE 2:** Unique locations of clinically significant prostate cancer (Gleason grade group ≥ 2) by transperineal mapping biopsy and mpMRI lesions of PI‐RADS 3–5

Quadrant Location	csPCa	Mean number positive cores	Range	csMRI
Right anterior	0	0		2
Left anterior	0	0		2
Right posterior	4	8.8	2–29	17
Left posterior	3	10.7	4–29	18
Right and left anterior	0	0		1
Right and left posterior	10	10	4–29	8
Right unilateral	5	4	2–6	20
Left unilateral	4	13.5	9–17	20
Right anterior and left posterior	0	0		2
Left anterior and right posterior	1	8.3	5–11	3
Right and left anterior and right posterior	0	0		0
Right and left anterior and left posterior	3	11	6–19	0
Right anterior and right and left posterior	3	11	6–19	1
Left anterior and right and left posterior	5	8.3	5–11	1
All 4 quadrants	2	9.6	2–29	0
Total clinically significant cases	32	9.6	2–29	57

*Note*: For example, right or left sided hemi‐ablation could be applied to in 9/32 (21.8%), whereas right and left posterior ablation could be offered to 10/31 (31.3%).

Abbreviations: csPCa, clinically significant prostate cancer; mpMRI, multiparametric magnetic resonance imaging.

**TABLE 3:** Comparison of csPCa to csMRI in men with PI‐RADS designation of 3–5

Quadrant	Sensitivity	Specificity	PPV	NPV	Accuracy	AUC (*p* value, 95% CI)
Entire prostate	81.3%	35.4%	45.6%	73.9%	53.8%	0.583 (0.209, 0.457–0.709)
Right side	17.4%	76%	10%	95%	62.5%	0.580 (0.551, 0.307–0.853)
Left side	25%	75%	5%	95%	75.2%	0.500 (1.0, 0.208–0.792)
Right anterior	11.1%	92.9%	22.2%	90.4%	85%	0.576 (0.460, 0.361–0.791)
Left anterior	18.2%	86.7%	25%	87.5%	81.3%	0.547 (0.615, 0.354–0.740)
Right posterior	48%	65.5%	38.7%	73.5%	60%	0.567 (0.337, 0.43–0.705)
Left posterior	51.9%	69.1%	45.2%	74.5%	65%	0.599 (0.15, 0.466–0.732)

Abbreviations: AUC, area under the curve; csMRI, clinically significant magnetic resonance imaging; csPCa, clinically significant prostate cancer; NPV, negative predictive value; PPV, positive predictive value.

In the 13 men who underwent prostatectomy, there was no change in GG group in 10 (77.9%) when compared with the mapping biopsy results. No cases were upgraded, whereas there was a downgrade of 1 GG in 3 patients. These three cases remained clinically significant despite the downgrade; 58/60 men with PCa underwent treatment or active surveillance (Table 4); 10/13 who had radical prostatectomy had csPCa, whereas three men had high volume bilateral GG1 disease. Men receiving focal therapy had lower GG and fewer quadrants involved compared with definitive therapy.

**TABLE 4:** Treatment choice by Gleason grade group and number of quadrants involved. Compared with definitive treatment the 25 men who had focal therapy had lower Gleason grade group

Treatment	No.	Mean GG	95% CI	GG 1 only	1 Q	2 Qs	3 Qs	4 Qs
Brachytherapy	8	2.75	1.6–3.9	1	1	3	2	1
External beam	4	2.75	0.4–5.0	4	2	4	3	0
Prostatectomy	13	2.15	1.5–2.8	4	2	4	3	0
Focal therapy	25	1.68	1.3–2.1	14	3	4	4	0
Surveillance	8	1.0	1.0	8	0	0	0	0
Total	58	1.9	1.6–2.2	31	8	15	12	1
*p* value			0.008					0.05

*Note*: All active surveillance patients had GG 1. The 4 prostatectomy men with GG 1 had high volume disease (average 8 positive cores).

Abbreviations: GG, grade group. Q, quadrant.

## 4 DISCUSSION

This investigation determined that a TPMB following mpMRI provides regions (quadrants) of the prostate containing csPCa missed by PI‐RADS 3–5 criteria with consequent improvement in the patient selection for focal therapy. The ‘classic’ hemi‐ablation or ‘hockey‐stick’ ablation pattern often recommended for focal ablation may not be ideal based on the results of this study. The quadrants most involved with csPCa are the two posterior zones, which made up 31.3% of the potential cases. A unilateral ablation could be considered in nine men, while just the right or left posterior quadrants in seven (Table 2). Only two cases involved all 4 quadrants leaving 93.8% of the men with csPCa potential candidates for subtotal therapy. When assessing the value of mpMRI for accuracy in diagnosis csPCa for the entire gland, the data in the current study agree with that reported by Westphalen.^14^ He reviewed 3449 men across 26 centres and estimated PPV at 35%, values not substantially different for the PPV in current study of 45.6%. Chu et al. analysed 344 men on an active surveillance protocol^15^ and found that an overall NPV of mpMRI was 79.5%, again similar to the 73.9% reported in the current study.

Several investigators have documented the limitations of mpMRI in identifying smaller PCa lesions. Johnson et al. reported when mpMRI was compared with WMRP specimens, only 45% of all PCa lesions and 65% of csPCa were detected.^3^ The majority of missed lesions were ≤1 cm. Le et al. reported sensitivity of MRI compared with WMRP as 47% for all lesions and to 72% for GG ≥ 2, which compares favourably to the sensitivity analysis in the current study of 51.4–81.3%, implying TPMB may be a substitute for histopathology of WMRP.^13^ Patel et al. compared TPMB with mpMRI and found similar detection characteristics as in the current study where sensitivity was 81.3 versus 81.3%, specificity 32.2 versus 35.9%, PPV 38.2 versus 45.6% and NPV 76.9 versus 73.9%, respectively.^1^

Systematic and targeted biopsy performed by the transperineal route can improve identification of csPCa. Radtke et al. reviewed 755 men who had transperineal fusion biopsy of mpMRI‐suspicious lesions followed by transperineal saturation biopsy (TSB, median 24 cores).^14^ The combination detected 97% of all csPCa lesions and was superior to targeted biopsy alone (*p* < 0.001).^14^ Ting et al. compared 148 patients who underwent MRI/US‐fusion and systematic biopsy with 80 patients with fusion biopsy plus TPMB (24 cores). The detection rate for the combined biopsy strategy improved detection of csPCa to 49% versus 40% (*p* = 0.02).^8^

The benchmark for validating the accuracy of a PCa detection protocol is the prostatectomy specimen. Alshak et al. performed fusion biopsy on 140 men and found 9/17 (52.9%) of the biopsies were upgraded from GG 1 to 2 compared to the prostatectomy specimens while 10/60 (16.7%) were downgraded from GG 2 to 1.^15^ In men with GG 1 PCa diagnosed by TRUS, a confirmatory biopsy, mpMRI and molecular classifiers can assist physicians in selecting patients for active surveillance. Kaye et al. analysed 1966 patients with GG 1 who had a negative confirmatory test and found upgrading to ≥GG 2 in 40% after prostatectomy.^16^ Although the number of patients who had prostatectomy in this investigation was small (*n* = 13), no patients were upgraded, and only one downgraded without a change in csPCa status.

Physicians pursuing focal therapy have utilised brachytherapy, HIFU, laser, electroporation, cryotherapy and radio frequency to create ablation zones.^17^, ^18^, ^19^ Most investigators have used mpMRI to both identify and target the treatment zone. Clinicians recognise that there is considerable uncertainty in identifying the boundary of the mpMRI detected lesions and utilise extensive margins or hemiablation to decrease the risk of leaving untreated areas. Unfortunately, the inability of mpMRI to identify out of field csPCa results in 50% failure rates in both modelling studies and clinical practice.^20^, ^21^ Confirmatory biopsy following focal therapy also demonstrates high out of field recurrence rates.^22^ The current study underscores the risk of limiting intraprostatic staging to ROIs identified by mpMRI when selecting patients for partial gland ablation.

The major limitation of this study was the relatively small sample size. However, the thorough interrogation of the prostate using a TPMB with a high biopsy density highlighted the benefit of incorporating this strategy after mpMRI to identify the regions of the prostate containing csPC. This study did not compare the TPMB to mpMRI plus 12‐core systematic biopsy. Although the latter does improve the detection of csPCa away from a PI‐RADS 3–5 ROI, it does not provide the precise localisation information generated from a thorough transperineal mapping procedure. There may also be a benefit to using a quadrant approach as opposed to lesions with a margin when considering partial gland ablation. This investigation did not address the costs associated with TRUS biopsy, TPMB and mpMRI. An outpatient TRUS is less costly than a TPMB, which has typically been performed in the operating room under anaesthesia, although new devices have been introduced allowing a TPMB to be done in the office with local anaesthesia.^23^ In either scenario, a prebiopsy mpMRI can be obtained, which can help guide the biopsy procedure if an ROI is present.

Of the 80 men who had a TPMB biopsy, 20 (25%) were negative. In contrast to an office TRUS biopsy where 30% of negative biopsies are false negatives (for csPCa) and need to be repeated, the likelihood of a false negative TPMB should be very low. Unless there is an increase in PSA or other clinical determinate, most of these men will not require a repeat biopsy.^24^ This is especially important for men with large glands. One patient had 169 biopsies taken at TPMB and had a prostate volume of 69 cc. His biopsy density was 2.4 and based on a recent publication that number could have been reduced to 1.5, which would have decreased the number of cores to 104.^10^ His PSAD was 0.87, and the mpMRI was negative. He also had a prior TRUS with one core containing Gleason GG 2. Another patient had 151 biopsies, but his biopsy density was 1.4. His PSAD was 0.019, and the mpMRI had a PIRADS 3 ROI. A prior TRUS was positive for Gleason GG 1. The TPMBs yielded no csPCa, and the men were placed on surveillance. In retrospect, as both men had very low PSADs and a low‐risk MRI, they could have been placed directly on surveillance without having had the TPMB.

This investigation did not directly compare TPMB with fusion biopsy. mpMRI‐based fusion biopsy with 12‐core systematic biopsy should be compared to a more extensive mapping in a randomised study. An investigation of this type could ascertain if there is a benefit to take biopsies for improved diagnosis of csPCa and to improve cancer localization for partial gland ablation. Validation of the latter could be accomplished by comparison with the radical prostatectomy specimens.

## 5 CONCLUSIONS

Transperineal mapping biopsy can enhance an mpMRI when considering partial gland ablation. It can aid in the selection of men for active surveillance by accurately excluding men with high grade disease. All the men who elected active surveillance in this investigation had Gleason GG 1 confirmed by TPMB. TPMB also accurately identified csPCa not detected by the MRI allowing the physician to correctly select the quadrants of the prostate that require ablation. This approach can increase the percent of men who are diagnosed with PCa for consideration of subtotal gland treatment. The most common portion of the prostate that qualifies for hemi‐ablation is both posterior zones.

## CONFLICT OF INTEREST

N. N. Stone and M. S. Lucia have ownerships in Triopsy, Inc. There was no financial assistance from Triopsy in this investigation or in the production of the manuscript.

## AUTHOR CONTRIBUTIONS

All authors contributed to the data collection, production of the manuscript and submission review.
